# Direct detection of alpha synuclein oligomers in vivo

**DOI:** 10.1186/2051-5960-1-6

**Published:** 2013-05-09

**Authors:** Hemi Dimant, Suneil K Kalia, Lorraine V Kalia, Liya N Zhu, Laura Kibuuka, Darius Ebrahimi-Fakhari, Nikolaus R McFarland, Zhanyun Fan, Bradley T Hyman, Pamela J McLean

**Affiliations:** 1 MassGeneral Institute for Neurodegenerative Disease, Department Neurology, Massachusetts General Hospital, Harvard Medical School, Charlestown, MA 02129, USA; 2 Division of Neurosurgery, Department of Surgery, Toronto Western Hospital, University of Toronto, Toronto, ON, CanadaM5T 2S8; 3 Movement Disorders Centre, Toronto Western Hospital, Division of Neurology, University of Toronto, Toronto, ON, CanadaM5T 2S8; 4 Institute of Anatomy & Cell Biology, Ruprecht-Karls University Heidelberg, INF 307, 69120, Heidelberg, Germany; 5 Department Neurology, Center for Translational Research in Neurodegenerative Disease, University of Florida, Gainesville, FL 32610, USA; 6 Department of Neuroscience, Mayo Clinic, Jacksonville, FL 32224, USA

**Keywords:** Parkinson’s disease, Alpha synuclein, Oligomers, Animal model, Live imaging

## Abstract

**Background:**

Rat models of Parkinson’s disease are widely used to elucidate the mechanisms underlying disease etiology or to investigate therapeutic approaches. Models were developed using toxins such as MPTP or 6-OHDA to specifically target dopaminergic neurons resulting in acute neuronal loss in the substantia nigra or by using viral vectors to induce the specific and gradual expression of alpha synuclein in the substantia nigra. The detection of alpha- synuclein oligomers, the presumed toxic species, in these models and others has been possible using only indirect biochemical approaches to date. Here we coinjected AAVs encoding alpha-synuclein fused to the N- or C-terminal half of VenusYFP in rat substantia nigra pars compacta and describe for the first time a novel viral vector rodent model with the unique ability to directly detect and track alpha synuclein oligomers ex vivo and in vivo.

**Results:**

Viral coinjection resulted in widespread VenusYFP signal within the nigrostriatal pathway, including cell bodies in the substantia nigra and synaptic accumulation in striatal terminals, suggestive of in vivo alpha-synuclein oligomers formation. Transduced rats showed alpha-synuclein induced dopaminergic neuron loss in the substantia nigra, the appearance of dystrophic neurites, and gliosis in the striatum. Moreover, we have applied in vivo imaging techniques in the living mouse to directly image alpha-synuclein oligomers in the cortex.

**Conclusion:**

We have developed a unique animal model that provides a tool for the Parkinson’s disease research community with which to directly detect alpha- synuclein oligomers in vivo and screen therapeutic approaches targeting alpha-synuclein oligomers.

## Background

Alpha synuclein (α-syn) aggregation has been closely linked to Parkinson’s disease (PD) etiology. Lewy Bodies, the pathological hallmark of PD are enriched with alpha synuclein fibrils [1,2] and point mutations and gene multiplications of the *SNCA* gene, which are associated with increased α-syn accumulation, result in familial PD [3-5]. Genome wide association studies have also demonstrated that single nucleotide polymorphisms in the *SNCA* loci may be a risk factor for idiopathic PD (Edwards 2010). Although the exact mechanism of α-syn induced toxicity remains unknown, recent observations allude to soluble α-syn oligomers being neurotoxic [6-9].

Rat models of PD have been developed based on the putative link between alpha synuclein and PD. First generation PD rat models employed neurotoxins such as 6-OHDA (6-hyroxydopamine) or MPTP (1-methyl-4-phenyl-1,2,3,6-tetrahydropyridine) which acutely degenerate dopaminergic (DA) neurons in the substanatia nigra (SN), but do not result in significant α-syn pathology [10]. Second generation models use viral vectors to target α-syn expression in the SN, resulting in a more gradual expression of α-syn with accompanying dopaminergic cell loss, which more closely resembles the chronic pathology of PD [11-14]. In these models and others, the detection of α-syn oligomers is based upon indirect approaches and biochemical techniques, but none offers direct detection of α-syn oligomers in vivo. In this study we present a third generation rat model of PD using bimolecular protein complementation assay (PCA) to enable the direct detection and visualization of α-syn oligomers along the nigrostriatal pathway. PCAs have been successfully applied to image protein-protein interactions *in vivo* based on a chemiluminescence signal from protein-luciferase conjugates, typically by transplanting immortalized cells expressing the protein-protein complementation pairs into organs of living mice [15]. While supporting the feasibility of complementation in vivo, none of the PCAs described to date have directly introduced each protein fragment separately and none have done so using fluorescence as an output rather than chemiluminescence.

The PCA approach, successfully applied in our laboratory to detect and image α-syn oligomers in vitro [7,8,16-18], is demonstrated here in vivo by viral mediated expression of human α-syn fused to either the N- or C- terminus half of venusYFP. Formation of fluorescently labeled α-syn oligomers is directly visualized along the nigrostriatal pathway ex-vivo in rat brain and in cortical neurons in vivo in a living mouse brain. Our novel approach for the direct detection of α-syn oligomers in vivo provides a powerful tool to study the role of α-syn oligomers in PD and to explore therapeutic approaches targeting α-syn oligomerization.

## Results

### Direct detection of alpha synuclein oligomers in the rat nigrostriatal pathway

**Figure 1:**
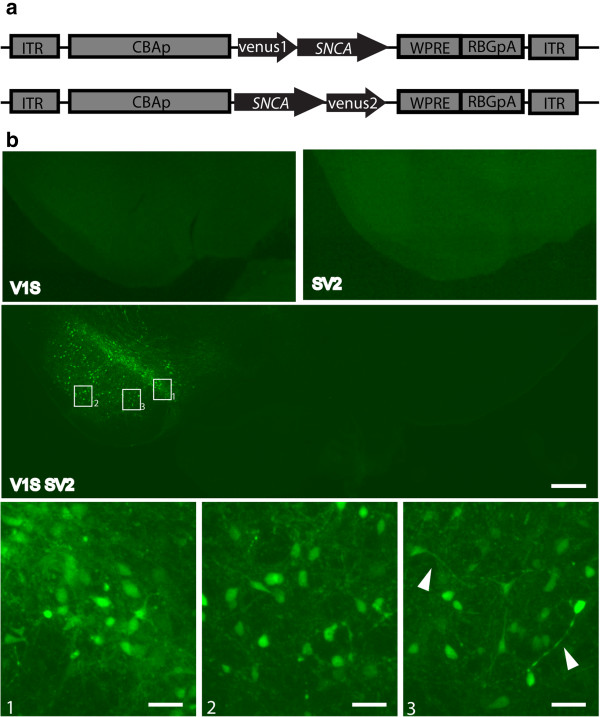
**Detection of alpha synuclein oligomerization in the SN using protein complementation assay.** Schematic representation of the AAV-V1S **(a, upper) **and AAV-SV2 **(a, lower)** constructs designed for the PCA. Coronal sections from the SN of rats injected with AAV-V1S and AAV-SV2 were mounted onto slides and directly imaged under a fluorescence microscope. Reconstituted VenusYFP is visible in animals injected with both AAV-V1S and AAV-SV2 and is localized to the SN **(b, middle panel)**. VenusYFP is visible in the SNpc and the SNpr within cell bodies and neurites **(b, lower panel)**. No fluorescence is observed in the non-injected contralateral side as well as in control animals injected with either AAV-V1S or AAV-SV2 **(b, upper panel)**. Scale bar 500 μm and 50 μm in middle and lower panels respectively.

In this study we developed two AAVs expressing human WT α-syn fused with either the N-terminus or C-terminus half of venusYFP protein (AAV-Venus1Syn and AAV-SynVenus2, referred to hereon out as V1S and SV2). AAVs were directly co-injected into the substantia nigra pars compacta (SNpc) of Sprague Dawley rats. Two additional control groups of animals were injected with either V1S virus or SV2 virus to exclude the possibility of non-specific fluorescence from one half of the venusYFP protein. At eight weeks post viral injection venusYFP fluorescence was clearly observable in the SN of animals injected with V1S + SV2, including the SN pars compacta and SN pars reticulata (Figure 1). Venus fluorescence was visible in cell bodies and in neuronal projections which, to some extent, could be traced to their respective neurons (Figure 1, 3 arrows). No fluorescence was visible in the non-injected contralateral side. The reconstitution of venusYFP fluorescence in vivo demonstrates the successful complementation of venusYFP halves via the formation of α-syn oligomers. Notably, in control animals injected with either V1S or SV2 alone we detected no venusYFP fluorescence in the injected (ipsilateral) side, demonstrating that the fragmented venus protein does not have any background fluorescence (Figure 1).

### Alpha synuclein associated toxicity

**Figure 2:**
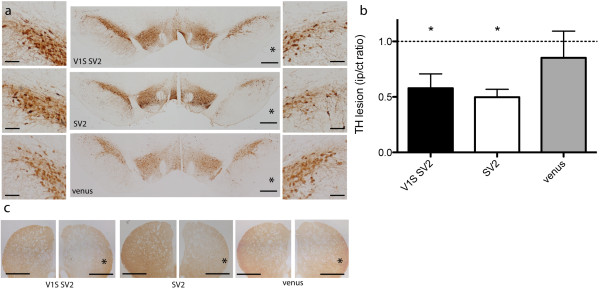
**Alpha synuclein associated neurotoxicity in the SN. **Unbiased stereological analysis of TH immunopositive cells in coronal sections across the SN was performed using DAB. Cytotoxicity is determined by extent of TH lesion as measured by the ratio of TH positive cells ipsilateral (right, asterisk) vs. contralateral (left) **(a)**. A 43% lesion was measured in rats injected with both AAV-V1S and AAV-SV2 (n = 8, p < 0.0001) and a 51% lesion was measured in rats injected only with SV2 (n = 7, p < 0.0001), both significantly lower than a non-lesion value of one **(b)**. The lesion, measured in the SNpc, was also noticeable in the striatum **(c)**. In the control group injected with full-length venusYFP a non-significant 15% lesion was measured (n = 4) **(b)**. Representative image are displayed. Scale bar 500 μm and 100 μm (zoomed-in images) in a and 500 μm in c.

To determine if the overexpression of α-syn oligomers the SN results in dopaminergic cell death we examined neuronal viability 8 weeks after viral injection. Coronal sections from the SN were immunostained for tyrosine hydroxylase (TH) and immunopositive cells visualized by DAB were counted using unbiased stereology both ipsilaterally and contralaterally (Figure 2a). The ratio of cells ipsilateral to contralateral was calculated and the extent of lesion was determined. Groups of animals injected with AAV8-V1S + AAV8-SV2 (n = 8) as well as control groups injected with AAV8-venus (n = 4) and AAV8-SV2 (n = 7) alone were included in the analyses.

Stereological analyses revealed 43% TH cell loss in animals co-injected with V1S + SV2 (Figure 2b). As expected a significant 51% TH cell loss was observed in the group of animals expressing only SV2, where oligomers are presumed to form but are not detectable (Figure 2b). A reduction in TH staining is also noticeable in the striatum, further conforming lesion formation (Figure 2c). In control animals expressing full length venus protein we observed an insignificant 15% TH cell loss consistent with the absence of a lesion (Figure 2b). These data demonstrates an α-syn induced toxicity resulting in neuronal cell loss.

### Direct visualization of alpha synuclein oligomers in the striatum

**Figure 3:**
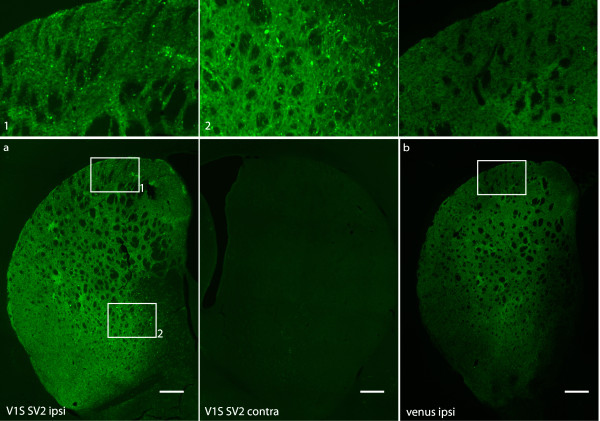
**Detection of alpha synuclein oligomers in the striatum. **The neuronal projections in the striatum of rats injected with AAV-V1S and AAV-SV2 in the SN were imaged for the presence of venusYFP fluorescence. Coronal sections from the striatum of rats injected into the SN were imaged under fluorescence microscope. VenusYFP is directly detected and localized to the striatum in animals injected with V1S and SV2, no fluorescence is detected contralateral **(a)**. VenusYFP fluorescence is also detected in the striatum of rats injected with AAV-venus **(b)**. Punctate fluorescence pattern is detected only in the striatum of animals injected with V1S and SV2 compared to the diffuse staining observed in animals injected with full length venusYFP (upper panel), suggestive for a possible accumulation of alpha synuclein within the striatum. Representative images are displayed. Scale bar 500 μm.

**Figure 4:**
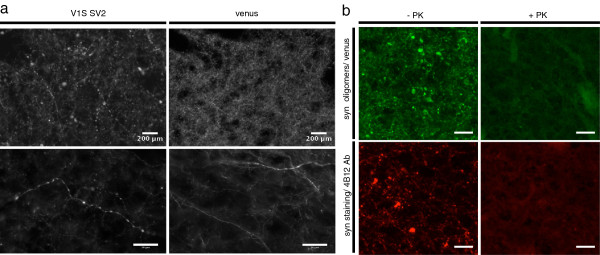
**Alpha synuclein associated pathology in the striatum. **Lower and higher magnification images of axons in the striatum of animals expressing V1S SV2 or full length venusYFP **(a)**. Beaded dystrophic axons are observed only in animals expressing α-syn compared to animals expressing venusYFP showing neurites with a normal appearance **(a)**. VenusYFP fluorescence and α-syn immunostaining were both diminished following PK treatment suggestive to the soluble nature of visualized oligomers **(b)**. Scale bar 200 μm upper panel **a **and 50 μm lower panel **a **and panel **b**.

The SN projects to and receives input from the striatum along the nigrostraital pathway. To determine if α-syn oligomers could also be detected in the striatum, coronal sections of rats injected with AAV8-V1S and AAV8-SV2 as well as from rats injected with AAV8-venus were mounted onto slides and directly imaged for fluorescence. Ipsilateral venusYFP fluorescence was visible in the striatum of animals injected with V1S + SV2, demonstrating the direct detection of α-syn oligomers in the striatum and confirming successful targeting of the viral injection (Figure 3a). No venusYFP fluorescence was detected in the contralateral side (Figure 3a). VenusYFP fluorescence was visible only in neuronal termini with no evidence of striatal cell body staining (Figure 3), arguing against retrograde transport of virus to the striatum. Of note, the pattern of venusYFP fluorescence in the striatum is distinctive to that observed in the SN. Venus fluorescence in the striatum of animals co-injected with V1S and SV2 is observed as multiple puncta (Figures 3a 1 and 2), which is not observed in the striatum of control animals injected with AAV8-venus (Figure 3b). Closer examination at higher magnification reveals beaded and dystrophic axons in animals expressing V1S and SV2 compared to healthy appearing axons in animals expressing full length venusYFP (Figure 4a), consistent with that reported in viral vector models [12] overexpressing α-syn. To determine if the observed α-syn oligomers represented insoluble aggregates we performed Proteinase K (PK) digestion. Following PK treatment, venusYFP fluorescence was completely absent, including puncta and neurites. α-Syn immunostaining using the antibody 4B12 was also decreased following PK treatment, suggesting the soluble nature of α-syn oligomers expressed in this model (Figure 4b).

### Neuroinflammation in the striatum

**Figure 5:**
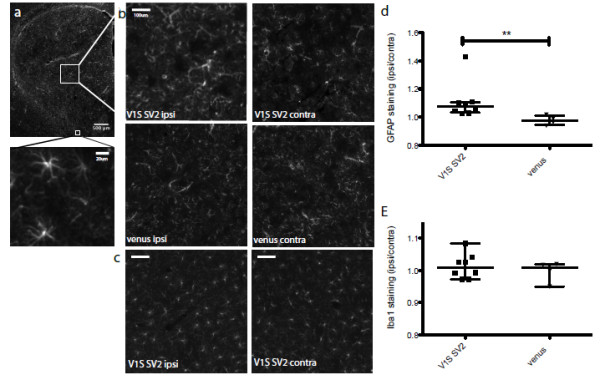
**Neuroinflammation in the striatum. **Striatal coronal sections were immunostained for GFAP expression **(a)**. Mean intensity quantification of GFAP **(b) **and Iba1 **(c) **staining was conducted in the striatum ipsilateral over contralateral, in the V1S and SV2 or venusYFP group. Data analysis revealed a 9% elevation in the ratio of GFAP expression ipsilateral over contralateral in animals injected with V1S and SV2 (n = 8, p < 0.005) compared to animals injected with full-length venusYFP (n = 4) showing no changes in the level of GFAP expression ipsilateral vs. contralateral **(d)**. Iba1 expression ratio in the V1S SV2 group was similar to the control group **(e)**. Representative images are displayed. Scale bar 500 μm and 20 μm in A, 200 μm in B and C.

Neuroinflammation plays an important role in the pathophysiology of PD [19] with studies linking striatal neuroinflammation to neurotoxicity and disease progression as a result of α-syn aggregation [20,21]. Striatal sections of injected animals were analyzed to determine whether the expression of α-syn oligomers results in a concomitant inflammatory response (Figure 5a). Animals injected with V1S + SV2 (n = 8) or full-length venusYFP (n = 4) were immunostained for GFAP, a marker of astrocyte activation (Figure 5b), and Iba1 a microglial marker (Figure 5c). High resolution image analyses revealed a significant elevation in the median expression of GFAP ipsilateral compared to the contralateral striatum of the V1S + SV2 group. In control animals expressing full length venusYFP, GFAP expression remained unchanged as reflected by a ratio value of 1 (Figure 5d). No difference was measured in the ratio of Iba1 expression with either the V1S + SV2 group or the venusYFP group (Figure 5e). The increase in GFAP expression in animals expressing α-syn suggests an α-syn associated gliosis in the striatum.

### In vivo imaging of alpha synuclein oligomers in the living mouse

**Figure 6:**
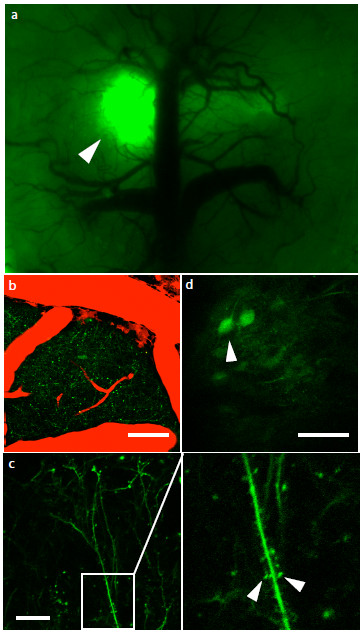
**In vivo imaging of alpha synuclein oligomers. **Mice injected intra-cortically with V1S and SV2 were installed with a cranial window and imaged to visualize alpha synuclein oligomerization in vivo. VenusYFP is visualized in the cortex and is localized to the injection site, demonstrating successful venusYFP complementation in vivo (**a**, arrow). Using two-photon microscopy venusYFP fluorescence is imaged showing neurites at 180 μm (**b**, green = venus, red = blood vessels) and cell bodies at 210 μm **(d)**. Higher magnification images reveal clear venusYFP fluorescence in dendrites **(c) **and in dendritic spines (**c**, arrows). Scale bar 50 μm.

The data thus far demonstrate our ability to detect α-syn oligomers along the nigrostraital pathway ex-vivo using a novel in vivo complementation assay of α-syn. Next we examined whether α-syn oligomers are detectable in the brain of a living animal using two-photon microscopy which, through deeper tissue penetrance, allows imaging of an intact brain up to 300 μm deep. C57bl6 mice were co-injected with AAV8-V1S + AAV8-SV2 into layer 2–3 of the cortex. After 4 weeks a craniotomy was performed to expose the brain and install a cranial window to allow imaging of the intact brain in the living animal [22]. Under low magnification and using an epifluorescence light source, venusYFP fluorescence could be clearly detected at the injection site in mice co-injected with V1S and SV2 (Figure 6a). Deep tissue imaging at higher magnification using two photon microscopy revealed venusYFP fluorescence along neurites (Figure 6b) at a depth of 160 μm (Figure 6c) and in cell bodies at a depth of up to 250 μm (Figure 6d).

## Conclusions

We herein present a novel in vivo protein complementation approach to directly detect α-syn oligomers in rodent brain. We demonstrate the successful targeted delivery of two AAVs into the substantia nigra (SN) and the direct ex-vivo imaging of α-syn oligomers in the nigrostriatal pathway including the SN and the striatum. We demonstrate α-syn induced toxicity as measured by a reduction in TH-positive neurons in the SN as well as gliosis (astrocytosis) in the striatum. We also demonstrate that multi-photon imaging can be applied to specifically image α-syn oligomers in the brains of living mice.

In recent years studies have shown that α-syn oligomers play a key role in PD etiology, shifting the blame from mature α-syn fibrils, such as in Lewy bodies, to soluble oligomers [6,23,24]. Previous studies conducted in our laboratory have shown the applicability of PCA to detect α-syn oligomers *in vitro* and have provided a means to further dissect the role of α-syn oligomers in PD [7,8,16-18]. Here we show for the first time the advantage of this powerful technique to detect and visualize α-syn oligomerization in vivo.

AAVs (V1S + SV2) were injected into the SN of Sprague Dawley rats and incubated for a period of 8 weeks, at the end of which histological analysis was conducted to validate the detection of α-syn oligomers. VenusYFP fluorescence is detected in the SN including the pars compacta (SNpc) and pars reticulata (SNpr) and to some extent in the ventral tegmental area (VTA). This demonstrates the direct delivery and expression of 2 viruses which results in in vivo α-syn oligomer formation. Control animals injected with only one of the viruses, either V1S or SV2, did not exhibit any fluorescence in the injection site, even though they presumably form oligomers as well [25]. Looking at neurons in the SN, we detect fluorescence in neurites, neuronal cell bodies, and the nucleus, as suggested by our inability to delineate nuclei [26]. Quantitation of tyrosine hydroxylase immunopositive neurons in the SN revealed an average lesion of 45% in animals expressing α-syn regardless of whether we could directly detect oligomers via fluorescence or not, in accordance with previously reported α-syn-induced lesions in viral vector rat models [11-14,27]. These results indicate that bimolecular fluorescent PCA with α-syn-Venus constructs does not artificially enhance α-syn toxicity, but rather is strictly an assay for α-syn oligomer formation. Furthermore, in animals expressing only full length venusYFP, no significant TH cell death was detected, demonstrating that neither the expression of full length venusYFP nor the injection procedure itself promotes a lesion. The ability to directly detect α-syn oligomers in vivo offers a powerful tool to elucidate the molecular mechanisms of α-syn mediated neurotoxicity and to examine α-syn based therapies.

Dopaminergic neurons project to the striatum along the nigrostraital pathway. Accordingly, we detect α-syn oligomers in the striatum of rats injected in the SN, with venusYFP fluorescence. This result demonstrates our ability to detect and visualize α-syn oligomers along the nigrostraital pathway but also confirms the successful targeting of the AAVs to neurons in the SN. Interestingly, in the striatum of rats expressing α-syn we observed distinct punctate fluorescence and dystrophic axons, compared to the diffuse venusYFP fluorescence and the healthy appearing axons observed in control animals expressing only venusYFP. The punctate staining, observed only in the striatum of α-syn expressing rats, was suggestive of α-syn aggregation, an intriguing observation in light of recent evidence demonstrating the synaptic localization of small α-syn aggregates in DLB patients, linking α-syn aggregation to synaptic pathology [28]. Proteinase K (PK) treatment reduced venusYFP fluorescence in the striatum including puncta and axons. α-Syn staining was likewise markedly decreased following PK treatment suggesting that α-syn accumulation in the striatum represent soluble α-syn oligomers.

Indeed this finding, taken together with accumulating evidence implicating α-syn in disease progression via a non-cell-autonomous neuroinflammatory process [29-31], led us to investigate whether inflammation occurs concomitantly with the aggregation of α-syn in the striatum and the presence of dystrophic neurites. Iba1 immunostaining showed no difference in microglial activation in response to V1S + SV2 expression or venusYFP control. However, GFAP analysis showed a small but significant elevation in GFAP expression ipsilateral in the V1S + SV2 group compared to that in control animals expressing only venusYFP. In accordance with our observation, asctrocytic gliosis is commonly reported in rodents models of α-synucleinopathies [32].

The uniquely designed PCA presented here has the advantage to detect and visualize α-syn oligomerization in post mortem tissue with minimal tissue processing, but could potentially be utilized to monitor the formation of α-syn oligomers over time in the brain of a living animal using two-photon microscopy. Recent advances in two-photon microscopy have enabled in vivo visualization of protein aggregation and neurodegeneration in the brain of an Alzheimer’s disease mouse model [22,33,34] and protein degradation in the brain of a PD animal model [35-37]. Here, using two-photon microscopy, we demonstrate the ability to image and detect α-syn oligomers in vivo. High-resolution images showed venusYFP fluorescence in neuronal cell bodies and axons, in agreement with a previous report examining cellular localization of GFP-tagged α-syn using two-photon microscopy [35,36]. We have also imaged α-syn oligomers in dendrites and dendritic spines, although we cannot rule out the possibility that the presence of a-syn in dendritic spines is an artifact of overexpression. Nonetheless, although α-syn is a pre-synaptic protein, our ability to image α-syn in the synapse may provide insight into the effects of α-syn on dendritic spines, which have been shown to degenerate in DLB patients [28] and thus may play a role in disease progression through trans-synaptic mechanisms. It is important to emphasize that multi-photon imaging of neurons in the brain of a living animal bestows the ability to perform longitudinal studies on individual neurons which, coupled with our ability to directly detect α-syn oligomers using fluorescence, offers a powerful tool with which to monitor α-syn aggregation and examine its direct effect on neurons over time.

Taken together, we present here a unique animal model to detect and monitor α-syn oligomer formation in the brain of rodents. Our animal model will give researchers the opportunity to better understand α-syn associated pathogenesis specifically in the context of α-syn oligomers in vivo but also offers great therapeutic screening potential for therapeutic agents that target α-syn oligomers.

## Methods

### Virus preparation

The viral vectors pAAV-CBA-VENUS1-SYNUCLEIN-WPRE (V1S) and pAAV-CBA-SYNUCLEIN-VENUS2-WPRE (SV2) were constructed by inserting the human *SNCA* gene, fused to either the N-terminus half of venusYFP (V1S) or the C-terminus half of venusYFP (SV2), into the EcoRV and NheI sites of the pAAV-CBA-WPRE vector [8]. pAAV-CBA-VenusYFP-WPRE was constructed by inserting the venusYFP gene into the XhoI and NheI sites of pAAV-CBA-WPRE vector. AAV serotype 8 was produced by the Harvard Gene Therapy Initiative as previously described [38].

### Injections

Sprague Dawley rats (300–350 gram) were anesthetized with intraperitoneal injection of ketamine/xylazine and placed in a stereotaxic frame. The surgical site was shaved and sterilized with betadine prior to making a 2 cm incision along the midline. The scalp was exposed and a unilateral injection targeting the SN was preformed at coordinates AP -5.2, ML -2 and DV -7.4 with bregma as a point of reference. For each animal, a total volume of 2 μl of virus was injected at a rate of 0.2 μl/min using a microinjection pump and 10 μl Hamilton syringe with a 30-gauge needle. For V1S + SV2 group 1 μl of AAV8- V1S (8.3•10^12^ viral genome/ml) and 1 μl of AAV8-SV2 (8.7•10^12^ viral genome/ml) were injected; for the venusYFP group and the SV2 group, 2 μl of AAV8-venus (1•10^12^ viral genome/ml) and 2 μl of AAV8-SV2 were injected, respectively. At the end of injection the needle remained in place for 5 minutes before gradual removal.

For in vivo imaging, C57 mice were anesthetized with 2% isoflorane and placed in a stereotaxic frame above a heated blanket. The surgical site was sterilized with betadine and isopropanol prior to conducting a 1 cm incision along the midline. The scalp was incised and an injection targeting layers 2–3 of the cortex was preformed at coordinates AP -1, ML -1 and DV -0.7 with bregma as a point of reference. A total volume of 2 μl (1 μl V1S + 1 μl SV2) was injected at a rate of 0.1 μl/min using a microinjection pump and 10 μl Hamilton syringe with a 33-gauge needle. After 4 weeks a cranial window was installed as described previously [22]. All studies were performed with the approval of the Massachusetts General Hospital Animal Care and Use Committee and in compliance with the National Institute of Health guidelines for the use of experimental animals.

### Tissue processing

At 8 weeks post-injection, rats were deeply anesthetized and transcardially perfused with 0.01 M phosphate buffered saline (PBS, pH7.4, Sigma) followed by 4% paraformaldehyde in PBS. Brains were post-fixed for 24–72 hours and transferred to a solution of 30% sucrose in PBS. Frozen brain were sectioned at 40 μm on a sliding microtome and kept in a cryoprotectant solution (30% sucrose, 30% ethylene glycol in PBS).

### Immunostaining

Free-floating coronal sections were washed overnight in PBS to remove cryoprotectant. Unless otherwise stated, all steps were performed at room temperature with three 10 min washes in PBS-TX (0.3% triton X-100) between each step. For diaminobenzidine (DAB) staining sections were treated with 10% methanol and 3% H_2_O_2_ to inhibit endogenous peroxidases, permeabilized in PBS-TX for 30 min and blocked in 5% NGS in PBS-TX for 30 min. Sections were incubated with primary antibody rabbit anti- TH (1:10 K, Millipore) or mouse anti α-syn (4B12 1:1000, Signet) over night at 4°C followed by secondary goat anti rabbit- biotin or goat anti mouse-biotin (1:200 Jackson ImmunoResearch) for 1 hour at room temperature and either avidin-biotin (Vectastain kit) or streptavidin-alexa555 for 1 hour at room temperature. Sections were incubated with DAB (Vector laboratories) to visualize TH positive cells, rinsed in PBS, mounted on superfrost slides (Fisher scientific) and coverslipped (Permount, Sigma). For immunofluorescence, sections were mounted onto superfrost, coverslipped with vectashield (Vector laboratories) and kept at 4°C.

### Proteinase K

Striatal coronal sections were mounted onto slides and dried prior to rehydration in 0.5 M Tris buffered saline containing 0.05% Tween-20 (TBST, pH7.4). Digestion of mounted sections was carried out with 50 μg/ml proteinase K at 55°C for 120 min. Sections were post fixed with 4% PFA for 30 min at room temperature and washed with TBST. Immunostaining of treated and non-treated sections with mouse anti α-syn clone 4B12 was conducted as described in the immunostaining section.

### Microscopy and stereology

Fluorescence images were obtained on either a Zeiss LSM510 META confocal microscope with X20 magnification or a Zeiss Axio observer inverted microscope, X5 magnification. In vivo images of anesthetized mice were obtained using a Bio-Rad 1024ES multiphoton microscope mounted on an Olympus BX50WI upright microscope. Dopaminergic neuron analysis was performed with an Olympus BX51 microscope. At least 8 coronal sections per animal throughout the SN were analyzed to determine tyrosine hydroxylase cell loss by conducting unbiased stereology cell counting according to the optical fractionator principle [39] using the Olympus CAST stereology software. At least 4 coronal sections for GFAP and 3 for Iba1 per animal, were analyzed to determine the level of protein expression throughout the striatum using high definition images obtained by automated collection and stitching of X5 images using the MetaMorph software (MetaMorph). The striatum, ipsilateral and contralateral, was outlined and the mean intensity in the region of interest was determined using FIJI (NIH).

### Statistics

GFAP and Iba1 expression was analyzed using Mann Whitney test. Data is expressed as group median with interquartile range. TH cell count was analyzed using one tailed *t*-test. Data is expressed as group mean ± SD. Statistical analysis was performed using GraphPad Prism software.

## Competing interests

The authors declare that they have no competing interests.

## Authors’ contributions

HD participated in the design of the study, carried out the experiments, data acquisition and analysis, and drafted the paper. SKK and LVK participated in the design of the study, viral injection and tissue processing. LZ participated in immunoassays and tissue processing. LNK participated in viral injection and tissue processing. DEF participated in lesion analysis. NRM designed and set up the viral injection methodology. ZF participated in plasmid construction. BTH assisted in drafting the paper, PJM conceived the study, designed the protein complementation assay, and assisted in drafting the paper. All authors read and approved the final manuscript.
